# The effect of obesity on chronic diseases in USA: a flexible copula approach

**DOI:** 10.1038/s41598-023-28920-6

**Published:** 2023-02-01

**Authors:** Robinson Dettoni, Cliff Bahamondes, Carlos Yevenes, Cristian Cespedes, Javier Espinosa

**Affiliations:** 1 Department of Economics, Universidad de Santiago de Chile, Santiago, Chile; 2 School of Education and Social Sciences, Universidad Andres Bello, Santiago, Chile

**Keywords:** Diseases, Obesity

## Abstract

We analyze the effect of obesity on the incidence of hypertension, hyperlipidemia and diabetes in USA using a health production theoretical framework along with a bivariate flexible semi-parametric recursive copula model that account for endogeneity. In this approach, the effects of control variables are flexibly determined using additive predictors that allow for a variety of effects. Our findings suggest that there exist a positive and significant effect of obesity on the prevalence of all chronic diseases examined. In particular, after endogeneity is accounted for, the probability of having hypertension, hyperlipidemia and diabetes for obese individuals are, respectively, 35%, 28% and 11% higher than those under the obesity threshold. These findings suggest that lowering obesity rates could lead to significant reductions in the morbidity and mortality associated with these diseases.

## Introduction

Obesity is described as a disease in which an abnormal or excessive amount of fat accumulates in the body and that may pose a health risk^1^. This disease has grown to epidemic proportions, and it has nearly tripled since 1975^2^. In fact, 13% aged 18 and over were obese in 2016. Obesity prevalence in the United States (USA) was 38% in 2014, up from 32% in 2004. This represents a notorious growth if we consider the figures back in the 80‘s where only 15% of the population recorded obesity according to the WHO. Nowadays obesity in USA is an issue for more than 40% of the population, a concerning figure if we compare other OECD countries with an obesity average of 20%^3,4^.

There are many variables that can unravel the incidence of obesity worldwide as well as in USA. In particular, it is generally known that genetics can explain obesity in many cases^5,6^. Economic variables such as business cycle expansions^7,8^, participation of women in the job market^9^ and processed food availability^10,11^ are positively related with obesity. There is also evidence that behavioral variables may have an impact in the occurrence of obesity. In this line, factors such as lifestyles, work routines, absence of physical activity, anxiety and bad eating habits have been found to be associated with obesity as well^12–15^. Environmental variables may also play an important role when it comes to obesity. In particular, mass media influence, highly caloric traditional food, consumerism and the need for immediate satisfaction are fostering factors for this epidemic^16–19^. Population density and sociodemographic variables such as age, gender, schooling and income level have also shown a significant association with obesity^20–31^.

Regardless of whether obesity is considered as a disease or a behavioral disorder, there is a consensus that it represents a major risk factor for chronic diseases in which hypertension, hyperlipidemia and diabetes can be found^32–39^. Individuals from various social strata in the United States have reported suffering from at least one of the chronic ailments stated, negatively impacting the country’s health system e.g.,^10,11,40–51^.

Obesity, which can lead to mortality and other morbidities, is responsible for a wide range of costs that governments must bear in terms of public health around the world^52–56^. In the USA for example, 61% of the costs of type 2 diabetes can be attributable to obesity and more than $100 billion dollar are destinated to deal with obesity and its effects such as cancer, gall-bladder problems, hypertension and other similar malignities^44,57–62^.

However, despite strong evidence of a link between obesity and the occurrence of chronic diseases, literature on its causal effect is still scarce. From an economic perspective, obesity, rather of being a single input into the health production function, can be considered as a possibly endogenous variable impacted by other health production variables. In addition, it seems possible to hypothesize that common unobserved factors simultaneously influence the propensity for obesity and the prevalence of chronic diseases.

This paper seeks to analyze the effect of obesity on the incidence of diabetes, hypertension and hyperlipidemia in USA using data obtained from the Medical Expenditure Panel Survey (MEPS), a health production framework and a flexible bivariate semi-parametric recursive copula model that account for endogeneity^63–65^. Unlike the traditional recursive bivariate model proposed by Heckman^66^ and used, for example, by Costa-Font J. Gil^37^, the semiparametric model is based on a copula structure^67,68^ allowing for different joint distributions and margins (logit, probit or cloglog-functions) for obesity and a set of selected chronic diseases analyzed in this work separately. Furthermore, in our research, the effects of continuous variables were estimated in a non-parametric form via spline functions. This is crucial to properly model the complex effects of variables such as education, age and income as they represent productivity and life-cycle factors that could affect obesity and each of the diseases non-linearly. If these relationships are not properly modeled then the effect of obesity on the probability of suffering a chronic disease (hypertension, hyperlipidemia and diabetes) may be biased^65^.

After applying the semiparametric copula model, our findings indicate that obesity has a positive and significant effect on the prevalence of each chronic diseases examined in this research. In particular, after endogeneity is accounted for, the estimated sampling average treatment effect for hypertension, hyperlipidemia and diabetes were, respectively, 35%, 28% and 11%. These findings suggest that lowering obesity rates could lead to significant reductions in the morbidity and mortality associated with these diseases, resulting in cost savings for the health system and the country’s human capital.

The article is organized as follows. In the next section, we analyze the connection between obesity and health using a health production theoretical framework and the bivariate flexible semi-parametric copula model that controls for endogeneity is presented. In Section “Data analysis”, we estimate and analyze the effect of obesity on the prevalence of hypertension, hyperlipidemia and diabetes using USA data. Section “Conclusion” concludes the paper with a discussion.

## Methodology

### Health and body mass production

We study the connection between body mass and the prevalence of chronic diseases based on the theory of health production. Costa-Font J. Gil^37^, Contoyannis and Jones^69^, Leibowitz^70^ and Grossman^71^, are some of the key contributions to this field. The standard model assumes that people devote time and resources to the development of domestic products like health ($y_2$). If a person engages in sports, eats nutritious foods, and so on, this person may develop bodily fitness ($y_1$), which impacts the production of health. Consequently, the production of an individual’s health can be represented as follows:

1 $$\begin{aligned} y_2=y_2(y_1,{{\textbf {x}}}_2, \varepsilon _2), \end{aligned}$$

where the vector ${{\textbf {x}}}_2=(I,{{\textbf {z}}}_2)$. As a result, health is defined by the individual’s fitness ($y_1$), income constraints (*I*), other health production determinants (${{\textbf {z}}}_2$) and other unobserved variables ($\varepsilon _2$). For obvious reasons, improvements in an individual’s fitness are expected to boost health care production, subject to the effects of other health production variables, whereas the effect of income determines an individual’s capacity to spend in health.

The production of individuals fitness level depends on individual’s income (*I*), other determinants (${{\textbf {z}}}_1$) and other unobserved variables ($\varepsilon _2$) such as the consumption of particular items like those produced at home, which contribute to the optimum level of fitness. Thus, the production of individuals fitness level can be written as

2 $$\begin{aligned} y_1=y_1({{\textbf {x}}}_1, \varepsilon _1), \end{aligned}$$

where ${{\textbf {x}}}_1=(I,{{\textbf {z}}}_1)$. As a result of (1) and (2), the empirical analysis of both health and fitness production is dependent on the identification of each variable’s individual effects. In this study, $y_2$ is defined in three different ways, each one of them representing the presence or absence of a chronic disease, from which we study hypertension, hyperlipidemia and diabetes. These are the main causes of avoidable mortality in USA e.g.,^10,33,53^. In turn, $y_1$ is represented by the presence or absence of obesity as a way of measuring individual fitness.

Since obesity is a potential endogenous variable influenced by other health production variables, the correlation between $\varepsilon _1$ and $\varepsilon _2$ is not expected to be zero. More specifically, lifestyle, psychological stress, as well as genetic and environmental factors could influence the predisposition for obesity and the prevalence of chronic diseases at the same time. To deal with the endogeneity of obesity, we propose a flexible bivariate semi-parametric copula model, which is presented in the next section.

### Semiparametric recursive bivariate copula model

There are basically two methods to deal with endogeneity in non-standard settings when it comes to instrument-based approaches, namely the simultaneous estimation and the two-stage technique. Regarding two-stage techniques, the simplest one is similar to linear two-stage squares and it is known as the control function approach^72,73^. Although the control function method is straightforward and fairly universal, it has issues when the endogenous variable is not continuous^74^. Simultaneous estimation methods are a second category of procedures that aim to create the complete joint distribution of the endogenous regressor and the outcome variable Zimmer^75^.

The recursive semiparametric copula additive model^65^ belongs to the family of simultaneous estimating methods, but it connects, via copula functions^67,68^, the two marginal distributions, producing a closed form equation for the likelihood function. This model is employed in this section to evaluate the impact of a binary endogenous variable on a binary outcome. A general explanation of identification, parameter estimation and the sampling average treatment effect is also provided. However, more specific details can be found in Radice et al.^65^, Marra et al.^64^ and Marra et al.^63^.

Since the key variables under study, obesity and the prevalence of chronic diseases (hypertension, hyperlipidemia and diabetes), are defined as dichotomous, a latent variable approach is employed to analyse the relationship between obesity and each one of the chronic diseases separately. As already mentioned in the previous section, obesity is a potentially endogenous variable, i.e., unobservable variables can affect both the inclination to obesity and the prevalence of a chronic disease in (1). Let us define $y^{*}_{1i}$ and $y^{*}_{2i}$ as the latent variables representing, respectively, obesity and the presence of a specific chronic disease. Thus, the model can be written as

3 $$\begin{aligned} y^{*}_{1i}&= \eta _{1i}({{\textbf {x}}}_{1i}) + \varepsilon _{1i} \end{aligned}$$

4 $$\begin{aligned} y^{*}_{2i}&= \gamma y_{1i} + \eta _{2i}({{\textbf {x}}}_{2i}) + \varepsilon _{2i} \end{aligned}$$

where, for $j=1,2$, $\eta _{ji}({{\textbf {x}}}_{ji})$ represents additive predictors (which will be discussed in the next section) and $\varepsilon _{ji}$ an error term. As these variables are not directly observable, we have:

5 $$\begin{aligned} y_{ji}={\left\{ \begin{array}{ll} 1, &{} \text {if}\ y^{*}_{ji}>0 \\ 0, &{} \text {otherwise}. \end{array}\right. } \end{aligned}$$

The joint cumulative distribution function (CDF) of the two variables is modelled using the parametric copula function $C:(0,1)^2 \rightarrow (0,1)$ e.g.,^63,64,67,68^ as

6 $$\begin{aligned} P(y_{1i}=1,y_{2i}=1)=C_{\theta }(P(y_{1i}=1),P(y_{2i}=1)). \end{aligned}$$

where $P(y_{ji}=1)=P(y^{*}_{ji}>0)=1-F_j(-\eta _{ji}({{\textbf {x}}}_{ji}))$, and $F_j(-\eta _{ji}({{\textbf {x}}}_{ji}))$ is the cumulative distribution function (CDF), which can be logit, probit or cloglog-functions. Therefore, the marginal CDFs are conditioned on covariates through $\eta _{ji}({{\textbf {x}}}_{ji})$. The association parameter $\theta$ describes the dependence between $y_{1i}$ and $y_{2i}$ after covariate effects at the marginal level are considered.

A key benefit of the copula approach is the simplicity with which a joint CDF can be produced by joining two arbitrary univariate marginal CDFs and a function *C*. In contrast to what is observed in traditional copula regression scenarios, in this work the binary variable $y_1$ appears as an explanatory variable in $F_2$, thus, the copula has a recursive structure. With respect to $y_2$, $y_1$ is endogenous due to the recursive structure if $\theta$ is statistically significant. The copula functions available in GJRM for practical modeling are listed in Table 1^76^. Additionally, Table 1 displays the relationship between $\theta$ and Kendall’s $\tau$-coefficient, which is a measure of nonlinear concordance dependence between two random variables that lies in the customary range $[-\,1,1]$. The Kendall’s $\tau$ for the Plackett copula (“PL”) is not shown in Table 1 since it is computed numerically as no analytical expression is available. Thus, Kendall’s $\tau$ is naturally built to capture the strength of dependence in copulas which is nonlinear in general, where traditional linear association measures fail (for example, Pearson correlation detects only linear dependence and it is not invariant to transformation of the marginal distributions)^67,77^.

Consider drawing two random pairs (*U*1, *V*1) and (*U*2, *V*2) from the joint distribution of *U* and *V*. Then the Kendall’s $\tau$-coefficient is defined as

7 $$\begin{aligned} \tau =P[(U_1-U_2)(V_1-V_2)>0]-P[(U_1-U_2)(V_1-V_2)<0]. \end{aligned}$$

Althought, Spearman’s rho is perhaps more popular within uncensored data due to its simplicity of its rank-based definition, Kendall’s tau usually gives the mathematically simpler derivation from a copula than Spearman’s rho, and has the clinical interpretation similar to the concordance index^78,79^. In addition, Kendall’s $\tau$ is invariant to any monotonically increasing nonlinear transformations of the marginal distributions *U* and *V*^77^.

The identification of the recursive copula model is the one obtained in^80^. In particular, two conditions need to be met. The copula function must exhibit first-order stochastic dominance with respect to $\theta$ in order to meet the first requirement. The presence of an instrument that influences the endogenous variable but not the outcome variable is the second requirement. However, the absence of this instrument permits to write down copula expressions with recursive structures e.g.^64,80–83^.

**Table 1:** Definition of the copulae implemented in GJRM, with corresponding parameter range of association parameter $\theta$ and relation between Kendall’s $\tau$ (which takes values in the customary range $[-\,1,1]$) and $\theta$.

Copula	$C_{\theta }(p_1,p_2)$	Range of $\theta$	Kendall’s $\tau$
AMH (“AMH”)	$\frac{p_1p_2}{1-\theta (1-p_1)(1-p_2)}$	$\theta \in [-\,1,1]$	$-\frac{2}{3\theta ^2}\left\{ \theta +(1-\theta )^2\log (1-\theta )\right\} +1$
FGM (“FGM”)	$p_1p_2\left\{ 1 + \theta (1 - p_1)(1 - p_2)\right\}$	$\theta \in [-\,1,1]$	$\frac{2}{9}\theta$
Plackett (“PL”)	$\left( Q-\sqrt{R}\right) /\left\{ 2(\theta -1)\right\}$	$\theta \in (0,\infty )$	−
Frank (“F”)	$-\theta ^{-1} \log \left\{ 1+(\exp \left\{ -\theta p_1\right\} -1) (\exp \left\{ -\theta p_2\right\} -1)/(\exp \left\{ -\theta \right\} -1) \right\}$	$\theta \in {\mathbb {R}}\backslash \left\{ 0\right\}$	$1-\frac{4}{\theta }\left[ 1-D_1(\theta )\right]$
Gaussian (“N”)	$\Phi _2\left( \Phi ^{-1}(p_1),\Phi ^{-1}(p_2);\theta \right)$	$\theta \in [-\,1,1]$	$\frac{2}{\pi }\arcsin (\theta )$
Student-t (“T”)	$t_{2,\zeta }\left( t_{\zeta }^{-1}(p_1),t_{\zeta }^{-1}(p_2);\zeta ,\theta \right)$	$\theta \in [-\,1,1]$	$\frac{2}{\pi }\arcsin (\theta )$

$\Phi _2(\cdot ,\cdot ;\theta )$ denotes the cumulative distribution function (cdf) of a standard bivariate normal distribution with correlation coefficient $\theta$, and $\Phi (\cdot )$ the cdf of a univariate standard normal distribution. $t_{2,\zeta }(\cdot ,\cdot ;\zeta ,\theta )$ indicates the cdf of a standard bivariate Student-t distribution with correlation $\theta$ and fixed $\zeta \in (2,\infty )$ degrees of freedom, and $t_{\zeta }(\cdot )$ denotes the cdf of a univariate Student-t distribution with $\zeta$ degrees of freedom. $D_1(\theta )=\frac{1}{\theta }\int _0^\theta \frac{t}{\exp (t)-1}dt$ is the Debye function and quantities *Q* and *R* are given by $1+(\theta -1)(p_1+p_2)$ and $Q^2-4\theta (\theta -1)p_1p_2$, respectively. The Kendall’s $\tau$ for “PL” is computed numerically as no analytical expression is available. Argument BivD of gjrm() in GJRM allows the user to employ the desired copula function and can be set to any of the values within brackets next to the copula names in the first column; for example, BivD = “N”. More details of the copula functions used in this research can be found, for example, in Marra et al.^64^ and Nelsen^67^.

### Additive predictor

This section provides a general explanation of the additive predictors used to model the endogenous and the outcome variables. More details can be found, for example, in Marra et al.^63^ and Dettoni et al.^84^. The key benefits of employing additive predictors are that they can handle a variety of covariate effects and that they may be calculated flexibly from the data without impossing parametric a priori forms. Let us consider a generic predictor $\eta _{\nu i}$ $\in {\mathbb {R}}$, and the overall covariate vector ${{\textbf {x}}}_{\nu i}$. The additive predictors for the endogenous and the outcome equations can be defined generically as

8 $$\begin{aligned} \eta _{\nu i}({{\textbf {x}}}_{\nu i})=\varphi _{\nu 0}+\sum _{k_\nu =1}^{K_\nu } f_{\nu k_\nu }({\textbf {x}}_{\nu k_\nu i}), \ i=1,\ldots ,n, \end{aligned}$$

where $\varphi _{\nu 0}\in {\mathbb {R}}$ is an overall intercept, ${{\textbf {x}}}_{\nu k_\nu i}$ denotes the $k_\nu$th sub-vector of the complete vector ${{\textbf {x}}}_{\nu i}$ (which contains, for instance, binary, categorical and continuous variables) and the $K_\nu$ functions $f_{\nu k_\nu }({{\textbf {x}}}_{\nu k_\nu i})$ represent generic effects which are chosen according to the type of covariate(s) considered. Each $f_{\nu k_\nu }({\textbf {x}}_{\nu k_\nu i})$ can be represented as a linear combination of $J_{\nu k_\nu }$ basis functions ${\mathcal {B}}_{\nu k_\nu j_{\nu k_\nu }}({{\textbf {x}}}_{\nu k_\nu i})$ and regression coefficients $\varphi _{\nu k_\nu j_{\nu k_\nu }}\in {\mathbb {R}}$, that is

9 $$\begin{aligned} f_{\nu k_\nu }({\textbf {x}}_{\nu k_\nu i})=\sum _{j_{\nu k_\nu }=1}^{J_{\nu k_\nu }} \varphi _{\nu k_\nu j_{\nu k_\nu }} {\mathcal {B}}_{\nu k_\nu j_{\nu k_\nu }} ({\textbf {x}}_{\nu k_\nu i}). \end{aligned}$$

As an example of basis functions, consider the B-spline basis. Assume that *J* denotes the number of B-spline bases. To define a *J* parameter B-spline basis, we first introduce a sequence of $J + D + 1$ knots $x^*_{\nu , 1}, x^*_{\nu , 2}, \dots , x^*_{\nu ,J+D+1}$ where the spline function is evaluated within the interval $[x^*_{\nu ,D+2}, x^*_{\nu ,J}]$. The B-spline basis is strictly local as each basis function is non-zero over the intervals between $D+1$ adjacent knots, where $D+1$ denotes the order of the basis. Therefore, B-spline basis functions are defined recursively as

$$\begin{aligned} {\mathcal {B}}^D_{\nu ,j}(x_{\nu })= \frac{x_{\nu }-x^*_{\nu ,j}}{x^*_{\nu ,j+D+1} -x^*_{\nu ,j}}{\mathcal {B}}^{D-1}_{\nu ,j}(x_{\nu }) + \frac{x^*_{\nu , j+D+2} -x^*_{\nu }}{x^*_{\nu ,j+D+2}-x^*_{\nu ,j+1}}{\mathcal {B}}^{D-1}_{\nu ,j+1}(x_{\nu }) \end{aligned}$$

and ${\mathcal {B}}^{D-1}_{\nu ,j}(x_{\nu })=1$ if $x^*_{\nu ,j} \le x_{\nu }< x^*_{\nu ,J+1}$ and 0 otherwise. Other formulations of basis functions are also feasible in (9) e.g.^85,86^.

Therefore, Eq. (8) can be written generically as

$$\begin{aligned} \eta _{\nu i} = \varphi _{\nu 0} + \sum _{k_{\nu } = 1}^{K_\nu } \varvec{{\mathcal {B}}}_{\nu k_\nu } ({{\textbf {x}}}_{\nu k_{\nu }i})^{\top } \varvec{\varphi }_{\nu k_\nu }, \end{aligned}$$

where $\varvec{{\mathcal {B}}}_{\nu k_\nu } ({{\textbf {x}}}_{\nu k_{\nu }i}) =\{{\mathcal {B}}_{\nu k_{\nu }1} ({{\textbf {x}}}_{\nu k_{\nu }i}),\dots ,{\mathcal {B}}_{\nu k_{\nu }J_{\nu k_\nu }} ({{\textbf {x}}}_{\nu k_{\nu }i}) \}^\top$ and $\varvec{\varphi }_{\nu k_\nu }= (\varphi _{\nu k_\nu 1},\dots , \varphi _{\nu k_\nu J_{\nu k_\nu }})^\top$. Furthermore, if $\varvec{{\mathcal {B}}}^{{\top }}_{\nu i} \varvec{\gamma }_{\nu } =\sum _{k_{\nu } = 1}^{K_\nu } \varvec{{\mathcal {B}}}_{\nu k_\nu } ({{\textbf {x}}}_{\nu k_{\nu }i})^{\top } \varvec{\varphi }_{\nu k_\nu }$, $\varvec{\varphi }_{\nu } =(\varphi _{\nu 0},\varvec{\varphi }_{\nu 1},\dots ,\varvec{\varphi }_{\nu K_\nu })^{\top }$ and $\varvec{{\mathcal {B}}}_{\nu i}=\{1,\varvec{{\mathcal {B}}}_{\nu 1} ({{\textbf {x}}}_{\nu 1 i})^\top ,\dots ,\varvec{{\mathcal {B}}}_{\nu K_\nu } ({{\textbf {x}}}_{\nu K_\nu i})^\top \}^{\top }$, we obtain

10 $$\begin{aligned} \eta _{\nu i} = \varvec{{\mathcal {B}}}^{ {\top }}_{\nu i} \varvec{\varphi }_{\nu }. \end{aligned}$$

Each $\varvec{\varphi }_{\nu k_\nu }$ has an associated quadratic penalty $\lambda _{\nu k_\nu } \varvec{\varphi }_{\nu k_\nu }^{\textsf{T}}\varvec{{\mathcal {B}}}_{\nu k_\nu } \varvec{\varphi }_{\nu k_\nu }$ that enables one to place particular properties on the $k_\nu$th function, such as smoothness. Note that each matrix $\varvec{{\mathcal {B}}}_{\nu k_\nu }$ only depends on the choice of the basis functions. Smoothing parameter $\lambda _{\nu k_\nu } \in [0,\infty )$ controls the trade-off between fit and smoothness, and as such it determines the shape of the related estimated smooth function. The overall penalty can be defined as $\varvec{\varphi }_\nu ^{\textsf{T}}\varvec{{\mathcal {D}}}_\nu \varvec{\varphi }_\nu$, where $\varvec{{\mathcal {D}}}_\nu =\textrm{diag}(0,\lambda _{\nu 1}\varvec{{\mathcal {D}}}_{\nu 1}, \ldots , \lambda _{\nu K_\nu }\varvec{{\mathcal {D}}}_{\nu K \nu })$. Smooth functions are typically subject to centering (identifiability) constraints (see Wood^86^ for more details). Several formulations of basis functions and penalty terms are feasible depending on the types of covariate effects considered e.g.^84,87^.

### Estimation, inferential specifics and sample average treatment effect (SATE)

Following, Radice et al.^65^, since $y_{1i}$ and $y_{2i}$ are binary variables taking values in $\{0,1\}$, we have four configurations of outcomes: $F(y^1_{1i}, y^1_{2i})=P(y_{1i}=1, y_{2i}=1)$, $F(y^1_{1i}, y^0_{2i})=P(y_{1i}=1, y_{2i}=0)$, $F(y^0_{1i}, y^1_{2i})=P(y_{1i}=0, y_{2i}=1)$ and $F(y^0_{1i}, y^0_{2i})=P(y_{1i}=0, y_{2i}=0)$. Let us define the complete vectors of parameters as $\varvec{\varphi } = (\varvec{\varphi }_1,\varvec{\varphi }_2, \theta )$. Then the log-likelihood function for the copula model can be expressed as

11 $$\begin{aligned} \ell (\varvec{\varphi })&=\sum _{i=1}^n[y_{1i} y_{2i}\log F(y^1_{1i}, y^1_{2i})+ y_{1i} (1- y_{2i})\log F(y^1_{1i}, y^0_{2i}) \nonumber \\&\quad + y_{2i} (1-y_{1i})\log F(y^0_{1i}, y^1_{2i}) + (1-y_{1i}) (1- y_{2i})\log F(y^0_{1i}, y^0_{2i})], \end{aligned}$$

where $$F(y^1_{1i}, y^1_{2i})= C(F_1(y^1_{1i}),F_2(y^1_{2i}), \theta )$$, $$F(y^1_{1i}, y^0_{2i})= F_1(y^1_{1i}) - C(F_1(y^1_{1i}),F_2(y^1_{2i}), \theta )$$, $$F(y^0_{1i}, y^1_{2i}) =F_1(y^1_{2i}) - C(F_1(y^1_{1i}),F_2(y^1_{2i}), \theta )$$ and $$F(y^0_{1i}, y^0_{2i})= 1-[F_1(y^1_{1i}) + F_2(y^1_{2i}) -C(F_1(y^1_{1i}),F_2(y^1_{2i}), \theta )]$$.

The modeling of binary data can be done with a great deal of flexibility thanks to our model specification. If an unpenalised estimation approach is employed to estimate $\varvec{\varphi } = (\gamma , \varvec{\varphi }_1,\varvec{\varphi }_2, \theta )$, then the resulting smooth function estimates are likely to be unduly wiggly e.g.^86^. Therefore, to prevent over-fitting, the following functions are maximized

12 $$\begin{aligned} \ell _p(\varvec{\varphi })= \ell (\varvec{\varphi }) -\frac{1}{2} \varvec{\varphi } ^{\textsf{T}}\Lambda \varvec{\varphi }, \end{aligned}$$

where $\ell _p$ is the penalized log-likelihood, $\Lambda =\textrm{diag}(\varvec{{\mathcal {D}}}_1,\varvec{{\mathcal {D}}}_2,1)$, and $\varvec{{\mathcal {D}}}_1$ and $\varvec{{\mathcal {D}}}_2$ are overall penalties which contain $\varvec{\lambda }_{1}$ and $\varvec{\lambda }_{2}$ defined as $\varvec{\lambda }_\nu =(\lambda _{\nu 1}, \ldots , \lambda _{\nu K_\nu })^{\textsf{T}}$ for $\nu =1,2$. The smoothing parameter vectors can be collected in the overall vector ${\varvec{\lambda }}=(\varvec{\lambda }_{1} ^{\textsf{T}},\varvec{\lambda }_{2}^{\textsf{T}})^{\textsf{T}}$. A robust and efficient trust region approach with integrated automatic multiple smoothing parameter selection is used to estimate the model parameters and smoothing coefficients. In this sense, the number of B-spline basis and knots are chosen automatically by minimizing the AIC criterion e.g.^63,64^.

Confidence intervals for any linear and nonlinear function of $\varvec{\varphi }$ are obtained from a Bayesian point of view, by recalling that the penalty term associated with the smooth functions of covariates represents the prior belief that these functions are likely to be smoother rather than wiggly. This implies setting an improper multivariate Normal prior on $\varvec{\varphi }$, which then leads to the posterior distribution $\varvec{\varphi }\sim {\mathcal {N}}(\hat{\varvec{\varphi }},[\varvec{{\mathcal {H}}}_p (\hat{\varvec{\varphi }})]^{\texttt {-}1})$, where $\varvec{{\mathcal {H}}}_p(\hat{\varvec{\varphi }})]$ is the model’s penalized Hessian. The rationale for using this result post-estimation is provided, for instance, in Marra and Radice^88^. They also show that using the above posterior distribution yields confidence intervals with better frequentist properties than those obtained using a frequentist approach itself. Other advantages of using the Bayesian result are that the distribution of nonlinear functions of $\varvec{\varphi }$ can easily be obtained by posterior simulation and that the resulting distribution need not be symmetric.

On the other hand, the effect of the treatment $y_{1i}$ on the probability that $y_{2i}=1$ is typically of primary interest. The purpose is to analyze how the endogenous variable (treatment) changes the expected outcome. As a result, the treatment effect is given by the difference between the expected outcome with treatment and the expected outcome without treatment. Different measures of treatment effect have been proposed in the literature. Here, we focus on the average treatment effect in the specific sample at hand (*SATE*), rather than that in the population^89^. In our case, following Radice et al.^65^, this can be defined as

13 $$\begin{aligned} SATE(\varvec{\varphi }, \varvec{{\mathcal {B}}})=\frac{1}{n} \sum _{i=1}^n [P(y_{2i}=1| y_{1i}=1)- P(y_{2i}=1| y_{1i}=0)], \end{aligned}$$

where $\varvec{{\mathcal {B}}} = (\varvec{{\mathcal {B}}}_{1i}, \varvec{{\mathcal {B}}}_{2i}, y_{1i})$ and $\varvec{{\mathcal {B}}}_{\nu i}=\{1,\varvec{{\mathcal {B}}}_{\nu 1} ({{\textbf {x}}}_{\nu 1 i})^\top ,\dots ,\varvec{{\mathcal {B}}}_{\nu K_\nu } ({{\textbf {x}}}_{\nu K_\nu i})^\top \}^{\top }$. Finally, $SATE(\varvec{\varphi }, \varvec{{\mathcal {B}}})$ can be estimated using $SATE(\hat{\varvec{\varphi }},\varvec{{\mathcal {B}}})$, whereas an interval for it can be obtained by employing Bayesian posterior simulation e.g.^63,64^.

## Data analysis

### Data and variables

The Medical Expenditure Panel Survey provided the data for this research (MEPS). Furthermore, the Agency for Healthcare Research and Quality, a division of the US Department of Health and Human Services, gathered and published them. The MEPS provides nationally-representative, micro-level information on medical spending, insurance status, and health conditions. In particular, we focus on the 2012 wave of the survey, where individuals aged between 18 and 64 years old were considered. Obesity is measured by the body mass index (BMI), defined as weight in kilograms divided by height in meters squared $(\text {kg/m}^{2})$. A person with a body mass index (BMI) above 30 is considered obese (WHO). Individuals who lacked all necessary socioeconomic and demographic control characteristics were not included in the sample (e.g., missing values for education or income). After exclusions, the final dataset contains 18,592 observations^65^. Table 2 summarizes the variables used in the analysis.

**Table 2:** Variables and results of the descriptive statistics.

Variables and Descriptive Statistics			
Variable	Definition	Mean	Std. Dev.
BMI	= Body mass index	27.861	6.195
Obesity	=1 if BMI > 30	0.295	0.456
Health	=1 Excellent, =2 very good, =3 good, =4 fair, =5 poor	–	–
Diabetes	=1 Diabetic	0.077	0.267
Hypertension	=1 Hypertension	0.249	0.432
Hyperlipidemia	=1 Hyperlipidemic	0.241	0.428
Limitation	=1 Health limits physical activity	0.080	0.271
Private	=1 Private health insurance	0.635	0.481
Age	= Age in years	39.891	13.459
Gender	=1 Male	0.470	0.500
Race	=1 White, =2 Black, =3 Native American, =4 others	–	–
Education	= Years of education	12.664	2.991
Income	= Income	62,498.98	53,732.80
Region	=1 Northeast, =2 mid-west, =3 south, =4 west	–	–

Data were obtained from the Medical Expenditure Panel Survey (MEPS) for USA. N = 18,592.

### Preliminary evidence

The means and standard deviations of the variables used in the empirical analysis are provided in Table 2. The existence of chronic diseases in our sample is of 24.9% with hypertension, 24.1% with hyperlipidemia and 7.7% with diabetes. 29.5% of adults are obese and the overall mean body mass index, BMI, is around 27.861 $\text {kg/m}^{2}$ (S.D. = 6.195). Certainly, the research backs up a growing body of evidence that certain populations are more vulnerable to certain diseases. In practice, people with hypertension account for only 5.6% of those under 60, while they are substantially more prevalent among those over 60. (57.76 %). Despite the fact that there were no statistical differences in hypertension between men and women, males (25.86%) are more likely than women to experience it (23.99% ). It is also worth mentioning that the prevalence of hypertension was higher among individuals who had no formal education or just completed primary school (32.25 % and 35.75%, respectively) than among those who completed secondary or high school (24.95% and 24.31%, respectively). When it comes to income, there are no significant differences in the proportion of people with hypertension between income quartiles, a phenomenon that also happens when the difference is measured by geographic area. We should also mention that black people (34%) and Native Americans (31.89%) have a greater prevalence of hypertension than whites (22.93%) and even other races (19.82%). The two other chronic conditions present a similar scenario. Hyperlipidemia is more prevalent in men (26.08%) than in women (22.40%). It is also prevalent in the senior population segment (55.65%). Surprisingly, those with a high or secondary educational level have a somewhat lower frequency of hyperlipidemia than those with a primary or zero educational level. Despite we may tend to think that quartiles with higher income have a slightly higher propensity to contract hyperlipidemia than those with lower income.

Similarly, little difference is seen in the portion of people with the disease when analyzed by race and region. As for diabetes, research shows that it increases with age, low educational level (primary or without education), and slightly for low-income-level (first and second quartile). Diabetes affects only 0.92 percent of people under the age of 30, whereas it affects 21.93 percent of people over the age of 60. Surprisingly, the diabetes rates for men and women are nearly the same.

Obesity is linked to specific variables such as gender, age, and income, among others. Obesity is more common in women (31.16%) than it is in men (27.61%). Obesity rates climb with age: 20.15% of those under 30 are obese, compared to 34.04% over 60. Obesity has a negative relationship with income, according to microeconomic data. Obesity affects more than 33% of individuals in the lowest income bracket, compared to only 23.69% of those in the highest income bracket. Education level is thought to influence body mass, and our findings appear to support this theory. Obesity is found to be negatively associated to education in our sample. Obesity affects 26.78% of those who have completed higher education, 31.55% of those who have completed secondary school, and 34.03% of those who have completed primary education. Also, 19.35% of the illiterate people are obese. Race is observed to play a role in obesity, as black and Native American people have a greater propensity to be obese (38.85% and 37.3% respectively) than whites or other races (29.04% and 11.96% respectively). Finally, in terms of geographic region, no great differences are observed in the propensity to obesity.

### Results

In this section several copula models with endogenous treatment are estimated. In particular, 54 models were fitted. Table 3 shows the best five models based on their Akaike information criterion (AIC) and Bayesian information criterion (BIC). First of all, the sampling average treatment effect (SATE) of obesity on hypertension, hyperlipidemia and diabetes is shown. Next, the measure of dependence is analyzed. Finally, the parametric and non-parametric effects are explained.

#### Estimated SATE

Tables 4, 5 and 6 show the results of utilizing the copula models outlined in Section “Semiparametric recursive bivariate copula model” to estimate the probability of an individual being obese as well as the prevalence of hypertension, hyperlipidemia, and diabetes. Tables are presented pairwise according to models (3) and (4). Since obesity is likely to be endogenous in equation (4) and for the identification of the copula model^80^, we use the individual’s physical limitation as instrument. We assume that this variable is redundant in that equation once obesity is considered. Besides, treatment regressions in Tables 4, 5 and 6 show that the instrument affects obesity once partial effects of the other variables have been considered. Therefore, this variable is a valid instrument for obesity.

Treatment equations in Tables 4, 5 and 6 also show that obesity had a statistically significant and positive effect on all chronic conditions studied, as expected. This is consistent with previous literature^37,38,47,50^. Furthermore, it is worth noting that the coefficients indicate significant heterogeneity in the specific impact of obesity, which, if not taken into account it could bias the results obtained.

The estimated SATE (in %) and confidence interval (CI) for the best five fitted copula models for each chronic disease are reported in Table 3. The chosen models show similar point estimates with overlapping CIs. Models not shown in the table show higher AIC / BIC support and systematically lower dependency than preferred models. Using the Plackett copula with probit-logit link functions combination, the estimated SATE of obesity on hypertension indicates that the probability of suffering hypertension increases by 35% for obese people compared to those who are not obese, fluctuating between 30.2% and 40.9% approximately.

The estimated SATE of obesity on hyperlipidemia indicates that the probability of suffering hyperlipidemia increases by 27.6% for obese people compared to those who are not obese, fluctuating between 21.9% and 35.5% approximately, the same copula and prior link functions combination were utilized for this. Regarding the SATE of obesity on diabetes, we use the Gaussian copula with logit-probit link functions combination, which indicates that the probability of suffering diabetes increases by 11% for obese people compared to those who are not obese, fluctuating between 6.7% and 15.6% approximately. These results can be compared with those obtained, for example, by Costa-Font and Gil^37^, who also found that obesity increases the probability of diabetes, hypertension and high cholesterol in Spain (43%, 47% and 20% respectively). Differences in the sizes of these effects between the two works can be explained, since in our approach different copulas functions were applied to model the joint distributions of obesity and each of the chronic diseases analyzed. Furthermore, in our research, the effects of continuous variables were estimated in a non-parametric form. This is crucial to properly model the complex effects of variables such as education, age and income as they embody productivity and life-cycle effects that are likely to influence obesity and each the of the diseases non-linearly. If these relationships are not properly modeled then the effect of obesity on the probability of suffering a chronic disease (hypertension, hyperlipidemia and diabetes) may be biased^65^.

We note that the SATE results do not differ greatly between the same copula with different link functions combination, but it does differ between the different copulas, hence, as explained by Marra et al.^64^ choosing the right copula model can have an impact.

**Table 3:** Estimated SATE (in %), Kendall’s $\tau$, AIC and BIC obtained using different copula models for the 2012 MEPS data.

Estimated SATE				
Copula (links)	$\widehat{\tau }$ (95% CIs)	$\widehat{\texttt {SATE}}$ (95% CIs)	AIC	BIC
*Hypertension*				
Plackett (p-l)	- 0.286(- 0.358,- 0.197)	35.0 (30.2,40.9)	37063.95	37385.13
Plackett (p-p)	- 0.288(- 0.382,- 0.216)	35.1 (28.4,41.0)	37064.19	37374.31
Frank (p-l)	- 0.263(- 0.339,- 0.200)	34.1 (28.5,39.3)	37065.46	37388.17
Frank (p-p)	- 0.263(- 0.336,- 0.178)	34.1 (27.4,40.6)	37065.77	37377.96
Student (p-p)	- 0.324(- 0.396,- 0.236)	35.9 (29.9,41.0)	37068.71	37379.42
*Hyperlipidemia*				
Plackett (p-l)	- 0.282(- 0.381,- 0.176)	27.6 (21.9,35.5)	37629.45	37973.19
Plackett (p-p)	- 0.281(- 0.366,- 0.195)	27.6 (19.9,34.4)	37629.75	37968.90
Frank (p-l)	- 0.247(- 0.330,- 0.157)	25.9 (19.2,32.1)	37631.58	37974.95
Frank (p-p)	- 0.247(- 0.345,- 0.149)	25.9 (20.3,32.6)	37631.86	37970.80
Student (p-l)	- 0.331(- 0.419,- 0.212)	29.2 (23.1,34.1)	37633.39	37977.53
*Diabetes*				
Copula (links)	$\widehat{\tau }$ (95% CIs)	$\widehat{\texttt {SATE}}$ (95% CIs)	AIC	BIC
Gaussian (l-p)	- 0.123(- 0.220,- 0.016)	11.0 (6.7,15.6)	29145.20	29465.59
Gaussian (l-l)	- 0.121(- 0.228,- 0.038)	10.9 (6.9,15.7)	29145.29	29490.05
Frank (l-l)	- 0.157(- 0.291,- 0.020)	12.3 (6.5,19.2)	29145.92	29490.77
Gaussian (p-p)	- 0.228(- 0.362,- 0.097)	16.1 (10.0,24.9)	29149.05	29436.31
Gaussian (p-l)	- 0.225(- 0.354,- 0.027)	15.9 (8.7,24.3)	29149.95	29445.81

95% confidence intervals for the SATE have been obtained using the method detailed in Section “Semiparametric recursive bivariate copula model”. For the link functions, the probit link is represented by p, while for the logit and cloglog links we use l and c respectively. For example, (l-p) refers to a logit link for the outcome equation (l) and a probit link for the treatment equation (p).

#### Parametric components

With endogeneity accounted for, the gender-specific effects are significant for either chronic diseases (Tables 4, 5 and 6). The difference can be seen in the lower probability of men being obese compared to women, yet a higher probability of acquiring any of the chronic diseases studied. In terms of the health levels indicated by the health variable, we find that those who have a poorer health status are much more likely to develop a chronic disease than people who have a better health status. As for race, there is a significant difference between the probability of being obese and also having hypertension or diabetes for black people and native american compared to white people. While this difference is not observed in the case of contracting hyperlipidemia, where only a significant difference is shown for black people compared to white people. Other races show significant and negative differences with respect to white people in each of the chosen copula models.

In the treatment equations (Tables 4, 5 and 6) we show the effect of the geographical area in which the person is located on the propensity to obesity. We note that, controlling for the north-east zone, there are significant and positive differences with those who live in the mid-west and south, which suggests that they are more likely to be obese than those who live in the north-est zone. While there is no significant difference with those located in the western zone.

In addition, we check the influence of having private health insurance on the likelihood of acquiring a chronic condition. According to the results, we note that there is a significant and positive difference in the probability of having hypertension and hyperlipidemia for those who have private health insurance. This suggests that people who contract a health insurance are more concerned about their health condition, effect that is not significant for the probability of having diabetes.

#### Non-parametric components

**Figure 1:**
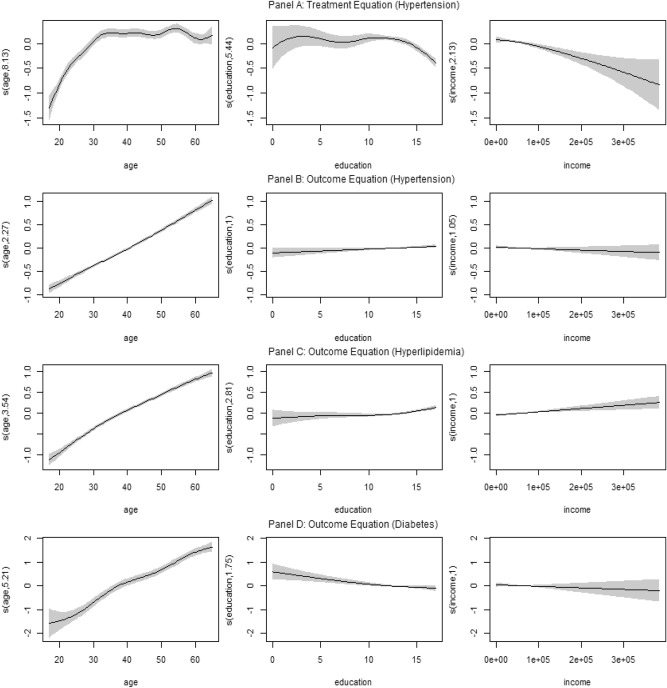
Smooth function estimates and associated 95 point-wise confidence intervals for obesity and all chronic diseases analyzed after applying the gjrm() function in GJRM to the MEPS data. The graph of the treatment equation (Obesity) is showed in Panel (**A**), while those for the outcome equations: Hypertension, Hyperlipidemia and Diabetes are showed in Panels (**B**), (**C**) and (**D**) respectively.

When using the different preferred models on the MEPS data, the smooth function estimates (age, education and income) for the treatment and outcome equations (and related intervals) are shown in Fig. 1. The estimated smooth functions obtained using the other copula models were similar.

The effects of age, education and income in the outcome equations show different degrees of non-linearity when comparing across different chronic diseases. This is also shown by the results of the treatment equations but at a higher degree of similarity, which was expected (aside from a few exceptions, such as the effect of education on the probability of having hypertension and the effect of income on the probability of having hyperlipidemia and diabetes).

Specifically, in Panel A (Fig. 1), we note that the effect of age on obesity, as a treatment for each of the diseases, is significant and positive between approximately 18 and 35 years of age. After age 35, it has an effect that tends to be constant until about age 55, with a slight decrease thereafter (for reasons of ease of explanation, one obesity regression (hypertension) is shown in Fig. 1 (Panel A), however, the non-parametric effect of obesity on hypertension is very similar to those for hyperlipidemia and diabetes). Regarding the effect of age on hypertension (Panel B), an increase in the propensity to have this disease is observed, which tends to be linear in the observed range. On hyperlipidemia (Panel C), a positive effect of age is observed in the observed range, showing a slight decrease in its growth from 35 years onwards. Finally, we see that the effect of age on the propensity to have diabetes (Panel D) is positive for the different age levels, and a more diffuse effect is shown between 18 and 25 years of age than in the following years.

The effect of education on obesity (Panel A), as a treatment for different diseases, is significant, approximately, from 12 years onwards, where there is a decrease in the probability of being obese when at least the secondary level of education is completed. Regarding its direct effect on the propensity to develop chronic diseases, we note that it is linear and significant on hypertension (Panel B), despite showing a low impact at the different levels observed. Regarding hyperlipidemia (Panel C), we see that its effect is non-linear and significant in the section of 12 years of education or more, where, counterintuitively, we note that the probability of having obesity increases after finishing secondary education. Finally, it is observed that education has a significant effect on diabetes (Panel D) and that it tends to be linear, where people who have more academic training are less likely to contract this disease.

When we look at the effect of income on obesity (Panel A) as a treatment for chronic diseases, we see that it is both significant and negative at different income levels, implying that people with a higher income have more opportunities to improve their nutritional quality and, as a result, have a lower risk of being obese. In the estimation by confidence intervals, the effect of income on hypertension (Panel B) and diabetes (Panel D) is not significant, as it comprises nearly zero for all of its reported values. Effects that contrast from those shown with hyperlipidemia (Panel C), in which a higher income level is associated with a lower risk of developing the condition, which is significant and linear in its observed range.

These conclusions are confirmed for the *p*-values reported in the Tables 4, 5 and 6. As for the mentioned variables, the estimated effects have the expected patterns. For example, age is a significant determinant in both equations. The probability of being obese and suffering a chronic disease are found to increase with age.

The likelihood of being obese, as well as the likelihood of having a chronic disease, appear to be closely associated with education. Education is likely to be correlated with an improvement in socioeconomic status and therefore people can lead a more permissive life in terms of their health. Regarding the effect of education on the probability of having a chronic disease, we note that it is significant in each of them, despite not showing a non-linear effect on hypertension.

We note that income has a significant effect on obesity. This suggests that a better financial situation can help a person not being obese. This is in contrast to its influence on two of the three chronic diseases under investigation, where it is found that while income has no bearing on the likelihood of having hypertension or diabetes, it does have a bearing on the likelihood of having hyperlipidemia, observing that it increases the probability of suffering hyperlipidemia at the higher part of its scale. This suggests that income can be a good predictor to explain a decrease in the probability of having obesity, where a better level of income could improve the way people eat, while the probability of having a chronic disease is not seen directly affected by income level, except for hyperlipidemia only when income is high, where other factors could be playing a role as well.

#### Measure of dependence (Kendall’s $\tau$)

In Table 3, where results of different copula models are presented for hypertension, hyperlipidemia and diabetes, all of the Kendall’s $\tau$ are significant and negative, meaning that the error term of the outcome equation (3) is negatively associated with the error term of the treatment equation (4). This negative association is consistent with related findings in Costa-Font and Gil^37^. Nevertheless, it might also be expected that those unobserved variables that are captured in the error term of the model for obesity (4) are positively associated with the ones of the model for each chronic disease in model (3). A possible explanation for the negative sign is the existence of measurement error in variables. The baseline model assumes that the BMI captures obesity without error. If we assume that the BMI measures obesity with error, there would be a source of negative correlation between the errors and thus a negative $\tau$. For instance, if an individual attends the gym frequently and builds muscles, his/her BMI would be above the healthy threshold so obesity would be overestimated for this person. At the same time, people who frequently exercise have a lower probability of developing a disease. Therefore, for these individuals, the probability of the -measured- obesity would be high, and the probability of suffering other diseases is lower, thus generating a negative correlation between the errors in the treatment and outcome equations. Now, consider the case of an individual whose BMI is too low because he/she has an eating disorder. This individual would be classified as not obese according to the rule assumed by the literature and this paper regarding BMI. However, this individual would have a high probability of acquiring one of the diseases studied in this document, therefore inducing -again- a negative relationship between the errors in the outcome and treatment equations.

Therefore, in order to inspect the resulting negative association, several approaches were taken to study the robustness of the estimated negative sign, which are analyzed in the following discussion.

The treatment is obesity, a binary variable that is 1 when the body mass index (BMI) is 30 or greater and 0 otherwise. The estimated negative association between error terms could be a consequence of the definition of the scale of the treatment variable. Therefore, we explored not only alternative definitions of the binary response variable for the treatment equation (4) but also different subsets of data to obtain evidence for or against the negative sign of $\tau$.

Regarding the approach of using several alternative definitions, we focused our attention on thresholds chosen according to the limits of BMI intervals established by WHO that define obesity class I, II and III, middle points of obesity class I and II, and a BMI of 42.45 as a particular point of class obesity III. For each one of these thresholds, a new version of the treatment variable Obesity was constructed and their corresponding set of models (3) and (4) for each chronic disease were fitted. For all of these optional settings, the Kendall’s $\tau$ was negative again, observing evidence for its originally found negative sign.

Keeping BMI=30 as the threshold to define obesity, the other approach that was analysed was the one of removing from the data those observations at an extreme part of the scale of BMI. Excluding observations at the lower part of the scale of BMI (BMI<18.5) is of special interest because those people are considered underweight, and therefore an increase in their BMI might affect their health status positively, not negatively as it is for people who is at the higher part of the scale. Therefore, we consider only those observations for which an increase in BMI should affect their health status negatively. Consistently with previous results, the outcomes of this subset show a negative Kendall’s $\tau$ too. We also used the same approach with some other BMI values, following the same criteria of using limit values and middle points of different intervals that define classes of BMI, particularly those of normal weight and overweight, for which we found negative Kendall’s $\tau$ again for every chronic disease. Similarly, we explored exclusions of observations in the higher part of the BMI scale, particularly for obesity class II and III, but without different results in terms of the sign of Kendall’s $\tau$. Therefore, not only alternative definitions of obesity but also different subsets of data provide results that do not shift the negative sign of Kendall’s $\tau$ into positive.

**Table 4:** Estimated coefficients and standard errors of the parametric and non-parametric components of the Plackett copula (PL) for the treatment and outcome equations for hypertension.

**Hypertension**				
Treatment equation				
Variable	Estimate	Standard error	Z-value	*P*-value
intercept	- 1.070	0.048	- 22.345	$$\texttt {0.000}^{{***}}$$
region2	0.200	0.057	3.531	$$\texttt {0.000}^{{***}}$$
region3	0.207	0.050	4.136	$$\texttt {0.000}^{{***}}$$
region4	0.053	0.055	0.958	$\texttt {0.338}$
gender1	- 0.127	0.034	- 3.791	$$\texttt {0.004}^{{***}}$$
race2	0.377	0.042	8.904	$$\texttt {0.000}^{{***}}$$
race3	0.289	0.157	1.842	$\texttt {0.066}^{{*}}$
race4	- 0.958	0.080	- 11.958	$$\texttt {0.000}^{{***}}$$
limitation	0.742	0.057	13.095	$$\texttt {0.000}^{{***}}$$

Variable	EDF	Ref.DF	Chi-square	*P*-value
s(age)	8.129	8.791	343.98	$$\texttt {0.000}^{{***}}$$
s(education)	5.437	6.499	76.60	$$\texttt {0.000}^{{***}}$$
s(income)	2.133	2.724	28.53	$$\texttt {0.000}^{{***}}$$

Outcome equation				
Variable	Estimate	Standard error	Z-value	*P*-value
intercept	- 1.782	0.035	- 50.484	$$\texttt {0.000}^{{***}}$$
obesity	1.248	0.096	12.971	$$\texttt {0.000}^{{***}}$$
health2	0.327	0.032	10.285	$$\texttt {0.000}^{{***}}$$
health3	0.538	0.033	16.234	$$\texttt {0.000}^{{***}}$$
health4	0.877	0.044	20.023	$$\texttt {0.000}^{{***}}$$
health5	1.091	0.066	16.652	$$\texttt {0.000}^{{***}}$$
private1	0.090	0.026	3.461	$$\texttt {0.000}^{{***}}$$
gender1	0.166	0.022	7.527	$$\texttt {0.001}^{{***}}$$
race2	0.259	0.031	8.259	$$\texttt {0.000}^{{***}}$$
race3	0.189	0.105	1.802	$\texttt {0.072}^{{ *}}$
race4	0.143	0.043	3.333	$$\texttt {0.000}^{{***}}$$

Variable	EDF	Ref.DF	Chi- square	*P*-value
s(age)	2.266	2.842	945.319	$$\texttt {0.000}^{{***}}$$
s(education)	1.000	1.000	4.211	$$\texttt {0.040}^{{**}}$$
s(income)	1.051	1.101	1.547	$\texttt {0.219}$

Kendall tau	Estimate	Confidence interval		
$\tau$	- 0.286	(- 0.358,- 0.197)		

95% for confidence intervals for $\tau$ have been obtained using the methods described in Section “Semiparametric recursive bivariate copula model”. The models were fitted using the functions gamlss() and gjrm()in GJRM by employing the “probit-logit” link functions combination. Furthermore, EDF and Ref.DF are the effective degrees of freedom and reference degrees of freedom of the non-parametric functions.

**Table 5:** Estimated coefficients and standard errors of the parametric and non-parametric components of the Plackett copula (PL) for the treatment and outcome equations for hyperlipidemia.

**Hyperlipidemia**				
Treatment equation				
Variable	Estimate	Standard error	Z-value	*P*-value
intercept	- 1.070	0.048	- 22.389	$$\texttt {0.000}$$ $$^{***}$$
region2	0.197	0.057	3.479	$$\texttt {0.000}$$ $$^{***}$$
region3	0.201	0.050	4.010	$$\texttt {0.000}$$ $$^{***}$$
region4	0.055	0.055	0.994	$\texttt {0.320}$
gender1	- 0.122	0.034	- 3.637	$$\texttt {0.000}$$ $$^{***}$$
race2	0.374	0.042	8.820	$$\texttt {0.000}$$ $$^{***}$$
race3	0.293	0.157	1.874	$\texttt {0.061}$ $^{*}$
race4	- 0.963	0.080	- 12.019	$$\texttt {0.000}$$ $$^{***}$$
limitation	0.746	0.057	13.162	$$\texttt {0.000}$$ $$^{***}$$

Variable	EDF	Ref.DF	Chi-square	*P*-value
s(age)	7.950	8.704	341.35	$$\texttt {0.000}$$ $$^{***}$$
s(education)	5.561	6.623	73.61	$$\texttt {0.000}$$ $$^{***}$$
s(income)	2.038	2.602	29.96	$$\texttt {0.000}$$ $$^{***}$$

Outcome equation				
Variable	Estimate	Standard error	Z-value	*P*-value
intercept	- 1.641	0.036	- 46.144	$$\texttt {0.000}$$ $$^{***}$$
obesity	0.995	0.116	8.541	$$\texttt {0.000}$$ $$^{***}$$
health2	0.273	0.031	8.861	$$\texttt {0.000}$$ $$^{***}$$
health3	0.431	0.033	13.247	$$\texttt {0.000}$$ $$^{***}$$
health4	0.755	0.044	17.019	$$\texttt {0.000}$$ $$^{***}$$
health5	0.804	0.064	12.498	$$\texttt {0.000}$$ $$^{***}$$
private1	0.157	0.026	5.970	$$\texttt {0.000}$$ $$^{***}$$
gender1	0.180	0.022	8.283	$$\texttt {0.000}$$ $$^{***}$$
race2	- 0.148	0.030	- 4.920	$$\texttt {0.000}$$ $$^{***}$$
race3	0.059	0.108	0.550	$\texttt {0.582}$
race4	0.170	0.041	4.096	$$\texttt {0.000}$$ $$^{***}$$

Variable	EDF	Ref.DF	Chi-square	*P*-value
s(age)	3.539	4.402	924.31	$$\texttt {0.000}$$ $$^{***}$$
s(education)	2.809	3.511	28.88	$$\texttt {0.000}$$ $$^{**}$$
s(income)	1.000	1.000	11.34	$$\texttt {0.000}$$ $$^{***}$$

Kendall tau	Estimate	Confidence Interval		
$\tau$	- 0.282	(- 0.381,- 0.176)		

The models were fitted using the “probit-logit” link functions combination. More details are given in Table 4.

**Table 6:** Estimated coefficients and standard errors of the parametric and non-parametric components of the Gaussian copula (N) for the treatment and outcome equations for diabetes.

**Diabetes**				
Treatment equation				
Variable	Estimate	Standard error	Z-value	*P*-value
intercept	- 0.666	0.029	- 23.203	$$\texttt {0.000}^{{***}}$$
region2	0.133	0.034	3.881	$$\texttt {0.000}^{{***}}$$
region3	0.122	0.031	4.015	$$\texttt {0.000}^{{***}}$$
region4	0.051	0.033	1.553	$\texttt {0.120}$
gender1	- 0.065	0.020	- 3.233	$$\texttt {0.001}^{{***}}$$
race2	0.231	0.026	8.952	$$\texttt {0.000}^{{***}}$$
race3	0.176	0.096	1.830	$\texttt {0.067}^{{*}}$
race4	- 0.541	0.043	- 12.534	$$\texttt {0.000}^{{***}}$$
limitation	0.456	0.036	12.742	$$\texttt {0.000}^{{***}}$$

Variable	EDF	Ref.DF	Chi-square	*P*-value
s(age)	5.369	6.498	359.25	$$\texttt {0.000}^{{***}}$$
s(education)	4.636	5.643	72.70	$$\texttt {0.000}^{{***}}$$
s(income)	1.948	2.483	33.64	$$\texttt {0.000}^{{***}}$$

Outcome equation				
Variable	Estimate	Standard error	Z-value	*P*-value
intercept	- 4.941	0.135	- 36.558	$$\texttt {0.000}^{{***}}$$
obesity	1.569	0.276	5.680	$$\texttt {0.000}^{{***}}$$
health2	0.620	0.130	4.781	$$\texttt {0.000}^{{***}}$$
health3	1.524	0.124	12.250	$$\texttt {0.000}^{{***}}$$
health4	2.037	0.136	15.033	$$\texttt {0.000}^{{***}}$$
health5	2.346	0.162	14.516	$$\texttt {0.000}^{{***}}$$
private1	0.084	0.070	1.199	$\texttt {0.230}$
gender1	0.138	0.060	2.294	$$\texttt {0.022}^{{**}}$$
race2	0.284	0.078	3.661	$$\texttt {0.000}^{{***}}$$
race3	0.708	0.236	2.997	$$\texttt {0.003}^{{***}}$$
race4	0.470	0.122	3.866	$$\texttt {0.000}^{{***}}$$

Variable	EDF	Ref.DF	Chi-square	*P*-value
s(age)	5.212	6.294	436.035	$$\texttt {0.000}^{{***}}$$
s(education)	1.749	2.191	15.425	$$\texttt {0.000}^{{***}}$$
s(income)	1.000	1.000	0.753	$\texttt {0.386}$

Kendall tau	Estimate	Confidence interval		
$\tau$	- 0.123	(- 0.220,- 0.016)		

The models were fitted using the functions gamlss() and gjrm()in GJRM by employing the “logit-probit” link functions combination. More details are given in Table 4.

## Conclusion

This paper aimed at addressing the effect of obesity on the incidence of diabetes, hypertension and hyperlipidemia in USA using a health production theoretical framework along with a bivariate flexible semi-parametric copula model that controls for endogeneity. Unlike traditional recursive probit models, the flexible copula model allows us for different joint distribution for the endogenous and outcome variables along with non-parametric estimation of the continuous control variables. Our findings imply that there is positive and considerable evidence of the effect of obesity on the prevalence of each chronic disease evaluated in this study after using the copula model. In particular, after controlling for endogeneity, the estimated sampling average treatment effect (SATE) for hypertension, hyperlipidemia and diabetes were, respectively, 35%, 28% and 11%. This shows that lowering obesity rates could result in significant reductions in the morbidity and mortality associated with these diseases, resulting in cost savings for the health system and the country’s human capital.

When it comes to obesity, our study encountered significant differences in sociodemographic terms. Regarding gender, obesity was more prevalent in women, therefore, public policies should also be gender oriented if countries want to win this battle against the burden of this disease. Age is positively related with obesity. Age sensitive measures and campaigns should be undertaken to encourage healthy habits in population. These two relevant results are in line with previous research. Now, our results suggest that income is negatively associated with obesity which contradicts other investigations. This finding is interesting since there could be a trend in people with higher incomes of allocating more resources on healthy food which can be prohibitive for people with lower incomes who are constantly encouraged to opt for fast and unhealthy food due to its availability and cost-effectiveness. Governments in the world should reach agreements and more flexibility to provide the markets with fresh fruits, vegetable and other natural products at a more convenient price, in order to reduce obesity rates in the population. There are also genetic factors associated with obesity which is evident in ethnics groups such as Afro-Americans and Native Americans. Awareness should be also risen as part of focalized public policies. The results provided in our research reported a statistically significant and positive effect of obesity on the prevalence of the three diseases in this study (diabetes, hypertension, and hyperlipidemia). Apart from confirming and strengthening previous research, given the amount of economic resources spent worldwide and its impact on the entire productive world, these findings should encourage better public strategies in dealing with obesity as an epidemic and a serious health concern. Countries that are able to contain this epidemic can reallocate finances to improve the quality of life and life expectancy of their citizens.

## Data Availability

The dataset analysed during the current study is freely available directly using the function data(meps), after loading the package GJRM in R (see Marra and Radice^76^).
